# Dietary Supplementation with Phytase and Protease Improves Growth Performance, Serum Metabolism Status, and Intestinal Digestive Enzyme Activities in Meat Ducks

**DOI:** 10.3390/ani10020268

**Published:** 2020-02-08

**Authors:** Junjie Jiang, Hao Wu, Dan Zhu, Jiameng Yang, Jianying Huang, Shuo Gao, Gang Lv

**Affiliations:** Institute of Livestock and Poultry, Tongwei Co., Ltd., Chengdu 610041, China; Jiangjj04@tongwei.com (J.J.); Wuh05@tongwei.com (H.W.); Zhud@tongwei.com (D.Z.); Yangjm01@tongwei.com (J.Y.); Huangjy02@tongwei.com (J.H.); Gaos04@tongwei.com (S.G.)

**Keywords:** meat ducks, growth performance, phytase, protease, low-energy and low-protein diet

## Abstract

**Simple Summary:**

Nowadays, as demand for reducing feed waste is increasing, it is imperative to apply enzyme preparations to achieve maximum effectiveness of feed. This study investigated the effects of dietary supplementation with phytase and protease in low-energy and low-protein diet on the growth performance, serum metabolism parameters, and intestinal digestive enzyme activities of meat ducks, which provided new insights into the positive roles of phytase and protease in growth performance, serum metabolism parameters, and intestinal digestive enzyme activities. This study contributes to the improvement in appropriate applications of exogenous enzyme preparations for poultry industry.

**Abstract:**

Two experiments were conducted to investigate the effects of dietary supplementation with protease and phytase on growth performance, serum physiochemical parameters, and activities of digestive enzymes in jejunal digesta of meat ducks. Experiment 1 was carried out to determine the effects of different protease or phytase on growth performance, serum physiochemical parameter, and activities of digestive enzymes in jejunal digesta of meat ducks to select the optimal phytase or protease. According to the hatching age and initial weight, a total of 5040 Cherry Valley ducks (15 days of age) were randomly assigned into six treatments. Treatments included a basal control diet (CON) and 5 basal diets supplemented with different enzyme preparations, which were phytase preparation A (PA, 160 g/t), phytase preparation B (PB, 800 g/t), protease preparation A (PTA, 80 g/t), protease preparation B (PTB, 300 g/t) and protease preparation C (PTC, 200 g/t). The enzyme activities were as follows: Phytase A and B as well as protease A, B, and C were 50,000, 10,000, 250,000, 50,000, and 60,000 U/g, respectively. Each treatment had 7 replicates with 120 meat ducks per replicate. Experiment 1 lasted for 28 days. The results showed that: compared with the CON group, the PA group significantly decreased contents of serum phosphorus and calcium (*p* < 0.05), and the PTA, PTB, and PTC groups had higher activities of trypsin in jejunal digesta (*p* < 0.05), and the activity of jejunal chymotrypsin in PTA group was greater (*p* < 0.05). Experiment 2 was carried out to determine the effects of dietary supplementation with protease and phytase in low-energy and low-protein diet on growth performance, serum physiochemical parameters, and activities of digestive enzymes in jejunal digesta of meat ducks. According to the hatching age and initial weight, a total of 5760 Cherry Valley ducks (15 days of age) were randomly assigned into four treatments on the basis of a trial of 2 × 2 factorial design. Treatments included a basal control diet (PC), basal diet supplemented with enzymes (PCE), low-energy and low-protein diet (LEP), and low-energy and low-protein diet supplemented with enzymes (LEPE), the nutrient levels of energy and CP of basal diet were 2747.2 cal·ME/kg and 16.80%, respectively, and the nutrient levels of energy and CP of low-energy and low-protein diet decreased 45.90 kcal·ME/kg and 0.52% on the basis of basal diet, respectively. According to the results of experiment 1, phytase A and protease A were determined as the optimal enzyme combination of Experiment 2, and additional dosage of which were identical with Experiment 1. Each treatment had 6 replicates with 240 meat ducks per replicate. Experiment 2 lasted for 28 days. The results showed that: compared with PC and LEP groups, PCE and LEPE groups had higher final weight and average daily gain (ADG) (*p* < 0.05), higher activities of trypsin and chymotrypsin in jejunal digesta (*p* < 0.05), lower contents of serum calcium and phosphorus as well as higher levels of high-density lipoprotein in the serum (*p* < 0.05). In conclusion, dietary supplementation with phytase and protease in different energy and protein diets could increase digestive enzymes in jejunal digesta, effect serum physiochemical parameters, improve metabolic status, and increase the growth performance of meat ducks. Meanwhile, with the dietary supplementation with phytase and protease in the lower energy and protein diet, the growth performance could reach to the degree of the higher energy and increased protein diet, but without the addition of phytase and protease.

## 1. Introduction

Phytate widely exists in plant feed and is abruptly stressful in the process of poultry’s digestion and absorption [1], since phytate could be combined with various nutrients (protein, fat, and minerals) in feed or digestive enzymes (α-amylase, trypsin, and pepsin) in animals’ gastrointestinal tract to form stable complexes [2], which in turn affects diverse aspects of function in the intestine, including lower activities of epithelial brush border enzymes and subsequent decrease in the digestibility of nutrients [3], thus reducing growth performance of poultry. Phytase, as a kind of common enzyme preparation in feed industry, was proven to be used as a growth-promoting agent on poultry [4], which has been repeatedly shown to have beneficial effects on removing chelation between phytic acid and protein in gastrointestinal tract of poultry [5]. Over the past two decades, the addition of exogenous phytases to poultry diets has become a standard practice. Recently, studies have further proposed that phytase could not only prevent excessive waste of nutrients in feed, but also played an essential role in reducing environmental pollution [4,5,6].

The ratio digestive tract to body length of poultry is shorter (about 4:1) compared with livestock, resulting in low endogenous digestive enzyme activity and sub-optimal nutrients digestibility [4]. Poultry enhancing or maintaining erepsin and trypsin activities in the gastrointestinal tract during the two weeks after hatching requires a long adaption period, which may result in serious economic losses to the poultry industry [7]. Moreover, low digestive enzyme activities in the gastrointestinal tract in turn affect diverse aspects of digestive function in the intestine of poultry, including intestinal inflammation, villous atrophy, and crypt hyperplasia [8]. Protease plays an important role in the food industry and has been used as a kind of enzyme addition for decades [9]. Concerned studies have previously demonstrated that dietary supplementation with exogenous protease could make up for the deficiency of endogenous protease and improve the digestibility of nutrients in feed as well as growth performance in poultry [10,11]. Meanwhile, protease might be helpful to hydrolyze anti-nutritional factors such as lectin and trypsin inhibitors, improving the utilization efficiency of amino acids in poultry [12].

In the livestock and poultry industry, the basic function of exogenous enzyme preparations is to improve the nutrition values of diets. In addition, dietary supplementation with exogenous enzyme preparation could also reduce the variability between the calculated and analyzed nutrient compositions [13]. Furthermore, dietary supplementation with exogenous phytase in low-energy diets or protease in low-protein diets has been proven to exert beneficial influences on the growth performance of poultry, including increases in feed intake and body weight [13,14,15]. China is the main production area of ducks, most of which are meat ducks, and there is a great waste of feeds for meat ducks because of insufficient endogenous enzyme secretions and subsequent poor digestibility of feeds. Although the various functions of exogenous phytase and protease have received extensive attention in broilers, there were few studies on how to appropriately apply in exogenous phytase and protease in feeds of meat ducks, and little is known about whether diets supplemented with exogenous phytase and protease in low-energy and low-protein diets could reach the degree of the a higher energy and increased protein diet, but without dietary supplementation with phytase and protease. Therefore, the objective of this study is to further evaluate the effects of dietary phytase and protease on growth performance, serum metabolism status, and intestinal digestive enzyme activities in meat ducks.

## 2. Materials and Methods

All procedures of animal experiments were carried out on the basis of protocols approved by the Animal Care Advisory Committee of Institute of Livestock and Poultry, Tongwei Co., Ltd., Chengdu, China (no. 20190610).

The phytase A (provided per gram of phytase 50000 units) and phytase B (provided per gram of phytase 10,000 units) were provided by DSM (China) Co., Ltd., Shanghai, China and Guangdong Yiduoli biology technolohy Co., Ltd., Zhuhai, China, respectively. The protease A (provided per gram of protease 250,000 units), protease B (provided per gram of protease 50,000 units) and protease C (provided per gram of protease 60,000 units) were also provided by Shanghai Nutritech Solution Co., Ltd., Shanghai, China, Beijing Smistyle Sci *&* Teah development Co., Ltd., Beijing, China, and Qingdao KDN biotech Co., Ltd., Qingdao, China, respectively.

### 2.1. Experimental Design and Animal Management (Exp. 1)

A total of 5040 fifteen-day-old Cherry Valley ducks (commercially purchased from Sichuan Guiliu Poultry Co., Ltd., Chengdu, China) was used in a 28-d experiment. At the beginning of the experiment, ducks were randomly assigned to 6 treatments with 7 replicate pens (60 males and 60 females per pen) on the basis of their initial body weight (BW) and sex. Treatments included a basal control diet (CON) and 5 basal diets supplemented with different enzyme preparations, which were phytase preparation A (PA, 160 g/t), phytase preparation B (PB, 800 g/t), protease preparation A (PTA, 80 g/t), protease preparation B (PTB, 300 g/t) and protease preparation C (PTC, 200 g/t).

All ducks were reared in cages (4.0 × 3.0 × 0.8 m) in a temperature-controlled room with a 24 h constant light schedule and had free access to water and feed throughout the experimental period. The basal diet (Table 1) was formulated to meet or exceed the nutrient requirements recommended by the National Research Council [16]. Diets were fed in pellet form, and the diameter of the pellets was 3.5 mm.

### 2.2. Sampling and Measurements (Exp. 1)

The ducks were weighed and feed intake was recorded on days 1 and 28, average daily gain (ADG), average daily feed intake (ADFI), and feed conversion ratio (FCR) were calculated. Dead ducks were collected, weighed, and recorded, and the mortality during the experiment was expressed as a percentage.

On day 29, prior to the morning feeding and following overnight fasting, 4 ducks (2 males and 2 females) with the average BW in each pen were chosen and bled. Blood samples were collected from the precaval vein into nonheparinized vacuum tubes. Briefly, after centrifugation (3500× *g* for 10 min at 4 °C), serum samples were collected and stored at −20 °C for serum parameters analysis. After bleeding, the same ducks were sacrificed by cervical dislocation, and the abdomen was immediately unfolded for the collection of gut sections. The entire small intestine was removed and cut into three segments: duodenum, jejunum, and ileum. Subsequently, 20 cm of jejunum was immediately isolated, which was gently squeezed to collect the jejunum digesta sample and stored at −20 °C for activity of digestive enzyme analysis.

Serum phosphorus and calcium levels were assayed using commercially available kits (Nanjing Jiancheng Bioengineering Institute, Nanjing, China), and the catalog numbers of which were C006-1-1 and C004-2-1, respectively. The jejunal digesta samples, were weighed, and then broken with an ultrasonic cell disruptor with the addition of 0.9% physiological saline in the ice bath. After centrifugation, the supernatant protein concentration was assayed using a protein quantification kit (Nanjing Jiancheng Bioengineering Institute, Nanjing, China) as the protein standard, and the catalog number of which was A045-2-2. Subsequently, activities of trypsin and chymotrypsin in the supernatant solution were analyzed using commercial kits (Nanjing Jiancheng Bioengineering Institute, Nanjing, China) according to the manufacturer’s instruction, and the catalog numbers of which were A080-2-2 and A080-3-1, respectively.

### 2.3. Statistical Analysis (Exp. 1)

Growth performance data were analyzed by ANOVA using the t-test procedure of SAS 9.0 software (SAS, Raleigh, NC, USA) with the pen being the experimental unit (*n* = 7). All other data were also analyzed by ANOVA using the *t*-test procedure of SAS 9.0 software (SAS Inst. Inc., Cary, NC, USA) with average data of 4 sampled ducks per pen as the experimental unit (*n* = 7). The results were presented as mean and standard error of means (SEM). Probability values less than 0.05 were considered significant, whereas probability values less than 0.10 was considered a tendency.

### 2.4. Experimental Design and Animal Management (Exp. 2)

A total of 5760 fifteen-day-old Cherry Valley ducks (commercially purchased from Sichuan Guiliu Poultry Co., Ltd., Chengdu, China) was used in a 28-day experiment on the basis of a trial of 2 × 2 factorial design. At the beginning of the experiment, ducks were randomly assigned to 4 treatments with 6 replicate pens (120 males and 120 females per pen) on the basis of their initial BW and sex. Treatments included a basal control diet (PC), basal diet supplemented with enzymes (PCE), low-energy and low protein diet (LEP) and low-energy and low-protein diet supplemented with enzymes (LEPE). According to the results of experiment 1, phytase A and protease A were determined as the optimal enzyme combination, and additional dosage of which were identical with experiment 1.

All ducks were reared in cages (6.0 × 4.0 × 0.8 m) in a temperature-controlled room with a 24 h constant light schedule and had free access to water and feed throughout the experimental period. The basal diet (Table 2) was formulated to meet or exceed the nutrient requirements recommended by the National Research Council [16], the nutrient levels of energy and CP of basal diet were 2747.2 cal·ME/kg and 16.80%, respectively, and the nutrient levels of energy and CP of low-energy and low-protein diet decreased 45.90 kcal·ME/kg and 0.52% on the basis of basal diet, respectively. Diets were fed in pellet form, and the diameter of the pellets was 3.5 mm.

### 2.5. Sampling and Measurements (Exp. 2)

All sampling and growth performance measuring procedures used were similar to those previously described for exp. 1.

Serum glucose, cholesterol, low-density lipoprotein, high-density lipoprotein, urea nitrogen, phosphorus and calcium levels as well as aspartate aminotransferase, alanine aminotransferase, and alkaline phosphatase activities were assayed using commercially available kits (Nanjing Jiancheng Bioengineering Institute, Nanjing, China), and the catalog numbers of which were F006-1-1, A111-1-1, A113-1-1, A112-1-1, C013-2-1, C006-1-1, C004-2-1, C010-2-1, C009-1-1, and A059-2-2, respectively. The jejunal digesta samples, were weighed, and then broken with an ultrasonic cell disruptor with the addition of certain saline in the ice bath. After centrifugation, the supernatant protein concentration was assayed using a protein quantification kit (Nanjing Jiancheng Bioengineering Institute, Nanjing, China) as the protein standard, and the catalog number of which was A045-2-2. Subsequently, activities of trypsin and chymotrypsin in the supernatant solution were analyzed using commercial kits (Nanjing Jiancheng Bioengineering Institute, Nanjing, China) according to the manufacturer’s instruction, and the catalog numbers of which were A080-2-2 and A080-3-1, respectively.

### 2.6. Statistical Analysis (Exp. 2)

Growth performance data were analyzed by two-way ANOVA using the Generalized Linear Models procedure of SAS 9.0 software (SAS, Raleigh, NC, USA) with pen as the experimental unit (*n* = 6). All other data were also analyzed by two-way ANOVA using the Generalized Linear Models procedure of SAS 9.0 software)(SAS, Raleigh, NC, USA) with average data of 4 sampled ducks per pen as the experimental unit (*n* = 6). The statistical model included the main effects of nutrient level, enzyme, and their interaction. The results were presented as mean and SEM. Statistical differences among treatment were determined by Duncan’s multiple-range test. For significance determination, the α-level was set as 0.05. Probability values less than 0.05 were considered significant, whereas probability values less than 0.10 was considered a tendency..

## 3. Results

### 3.1. Growth Performance (Exp. 1)

As shown in Table 3, no differences in growth performance were detected among ducks of the CON, PA, PB, PTA, PTB, and PTC groups (*p* > 0.10), but meat ducks fed diets supplemented with phytase or protease exhibited numerically higher ADG and ADFI than those fed the CON diet (*p* > 0.10).

### 3.2. Serum Physiochemical Parameters and Digestive Enzyme Activities (Experiment 1)

Table 4 presents the differences in serum physiochemical parameters and digestive enzyme activities of jejunal digesta among the 6 groups. Compared with the CON group, the PA group significantly decreased contents of serum phosphorus and calcium (*p* < 0.05), and the PTA, PTB, and PTC groups had higher activities of trypsin in jejunal digesta (*p* < 0.05), and the activity of jejunal chymotrypsin in PTA groups was greater (*p* < 0.05).

### 3.3. Growth Performance (Exp. 2)

The growth performance is given in Table 5. Dietary supplementation with phytase and protease had higher final weight and ADG compared with groups without supplementation of phytase and protease (*p* < 0.05), and there were interactive trend effects on ADG between nutrients levels and supplementation of enzymes (*p* < 0.10). In addition, dietary supplementation with phytase and protease tended to decrease FCR compared with groups without supplementation of phytase and protease (*p* < 0.10).

### 3.4. Serum Physiochemical Parameters and Digestive Enzyme Activities (Experiment 2)

As shown in Table 6, compared with groups without supplementation of phytase and protease, dietary supplementation with phytase and protease had higher serum contents of high-density lipoprotein as well as lower serum levels of phosphorus and calcium (*p* < 0.05), in addition, supplementation of phytase and protease decreased activities of trypsin and chymotrypsin compared with groups without supplementation of phytase and protease (*p* < 0.05). However, there were no interactive trend effects on serum physiochemical parameters and digestive enzyme activities between nutrients levels and supplementation of enzymes (*p* > 0.10).

## 4. Discussion

Recent studies have shown that dietary supplementation with phytase or protease had positive effects on growth performance of poultry via their specific action [12,17,18]. Shirley et al. (2010) have shown that phytase supplementation in diets improved growth performance in broilers [19]. Onyango et al. (2005) also reported that broilers fed diets supplemented with 1000 U/kg phytase showed improved body weight gain and ADFI [20]. Similar findings were observed by other researchers [21,22], and they reported that dietary supplementation with phytase could improve growth performance of broilers. Meanwhile, Angel et al. (2011) found that the addition of protease improved ADG and F/G of broilers [10]. Freitas et al. (2011) indicated that protease could improve F/G of broilers [23]. In the present study, although it showed no significant difference, meat ducks fed diets supplemented with phytase or protease exhibited numerically higher ADG and ADFI than those fed CON diet. Thus, phytase A and protease A were chosen as the optimal enzyme additives in Experiment 2 on basis of growth performance data from Experiment 1. Growth performance data from Experiment 2 revealed that the addition of phytate and protease combinations leaded to better ADG and final body weight of meat ducks, there was no published research on the effects of phytase and protease combinations on growth performance in meat ducks. The beneficial effects of phytase and protease combined supplementation on ADG and final BW of meat ducks might be associated with the synergistic effect of phytase and protease supplementation. In the present study of Experiment 2, with the dietary supplementation with phytase and protease in lower energy and protein diet, the growth performance could reach to the degree of the higher energy and protein diet but without the addition of phytase and protease, which was in line with the study of Akter et al. (2017) and Cowieson et al. (2019) that phytase and protease supplementation could enhance the feed efficiency and improve growth performance of poultry [24,25]. Starch and fatty acid are the main energy supply materials in livestock and poultry, which could chelate with phytate and reduce the energy utilization [26]. Farrell et al. (1993) reported that the addition of phytase could significantly improve the apparent metabolic energy of sorghum-soybean diet [27]. Farrell et al. (1998) similarly indicated that dietary supplementation with phytase could enhance the metabolic energy of meat duck diets [28]. Del Alamo et al. (2008) reported that dietary supplementation with protease could enhance the apparent total tract digestibility of protein in broiler chickens [29]. The phytate molecules of many crops are spherical, which usually are stored in protein-rich tissues (such as germ and aleurone layer), the solubilities of protein and phytate are very similar, resulting in strong chelation between globules of phytate molecules and protein in crops, the addition of phytase could prevent the excessive formation of protein-phytate chelates, even after the formation of protein-phytate chelates, the addition of phytase could also cooperate with pepsin to degrade the protein with maximum efficiency, phytase itself could not hydrolyze protein, so phytase should be combined with pepsin to improve the utilization of protein [30,31,32]. Thus, we suspected that phytate and protease supplementation had a synergistic effect in the gut due to the preferable digestibility and absorptive environment, which leaded to better growth performance in meat ducks.

The enzyme activities in digestive tract were considered as important factors that could influence intestinal health and nutrient digestibility [33]. Sufficient phytase and protease in the diets are important elements for the digestive system of poultry, as phytase supplementation could increase feed efficiency and gut health [27,28], while protease can affect the intestinal digestive enzymes activities and microbiota of digesta [34]. Protease could increase trypsin and chymotrypsin activities of broiler chicks [35], which was consistent with our results in Experiment 1. Meanwhile, in the present study of Experiment 2, phytase and protease combined supplementation in basal or low-energy and low-protein diet could both significantly increase trypsin and chymotrypsin activities of ducks in the jejunum. Thus, the increased trypsin and chymotrypsin activities by dietary supplementation with protease and phytate may have contributed to the improvement of nutrient digestibility by enhancing ducks’ digestive and absorptive function, thus improving growth performance of ducks. Non-starch polysaccharides enzymes (usually xylanase) and phytase can promote each other to improve the utilization of protein and energy in feed and the growth performance of poultry [36]. Phytic acid and water-soluble non starch polysaccharide can be closely combined in low-energy diet, which means that non-starch polysaccharide enzyme can more easily approach the surface of phytate and improve the degradation rate of phytate [36,37]. Thus, the two functions are complementary in concern respects, thus we suggested protease and phytate may have an interactive effect in the activities of trypsin and chymotrypsin in the intestinal digesta.

The chemical components in serum are mainly composed of the decomposition products in the gut and the metabolites released by tissue cells, which could reflect the accurate metabolic status of the body [38,39]. As serum high-density lipoprotein is directly related to metabolism of cholesterol, an enhancement in serum high-density lipoprotein could result in improved lipid metabolism [40]. In the present study, phytase and protease combined supplementation in basal or low energy and protein diet could significantly increase contents of serum high-density lipoprotein, this was in line with the study of Zeafarian et al. (2013), which indicated that addition of phytase could improve lipid metabolism of young broilers [41]. Similarly improved absorptive status of calcium and phosphorus in the body results in decreasing contents of serum calcium and phosphorus [42]. Conversely, the increasing contents of serum calcium and phosphorus means that the absorptive status of calcium and phosphorus in the body is worsen [43]. Previous studies reported that dietary phytase supplementation had lower contents of serum calcium and phosphorus in poultry [44,45], which was in line with our results in Experiment 1. Meanwhile, in the present study of Experiment 2, phytase and protease combined supplementation in basal or low-energy and low-protein diet could significantly increase contents of calcium and phosphorus in the serum, which may mainly result from the addition of phytase.

## 5. Conclusions

In conclusion, dietary supplementation with phytase and protease in different energy and protein diets could increase digestive enzymes in intestinal digesta, effect serum physiochemical parameters, improve metabolic status, and increase growth performance of meat ducks. Meanwhile, with the dietary supplementation with phytase and protease in lower energy and protein diet, the growth performance could reach to the degree of the higher energy and protein diet but without dietary supplementation with phytase and protease. Therefore, our results suggested that phytase and protease could be potential enzyme preparation combinations in low-energy and low-protein diet for improving growth performance, serum metabolism status, and intestinal digestive enzyme activities in meat ducks.

## Figures and Tables

**Table 1:** Compositions and nutrient contents of the experiment basal diets (air dry basis, %, Experiment 1).

Item	CON ^7^
Ingredients, %	
Maize	53.89
Wheat flour	4.50
Wheat bran	1.58
Rice bran	5.00
Soybean meal	13.00
Cottonseed meal	4.00
Maize germ meal	8.00
Feather meal	2.00
Distillers dried grains with solubles	3.00
Maize gluten meal	1.70
Calcium carbonate	1.64
Dicalcium phosphate	0.59
L-Lysine HCl	0.42
DL-Methionine	0.01
L-Threonine	0.02
NaCl	0.20
Enzyme preparation ^1^	0.04
Bacitracin methylene disalicylate premix ^2^	0.01
Preservatives ^3^	0.10
Vitamin premix ^4^	0.10
Mineral premix ^5^	0.20
Total	100.00
Calculated Nutrients ^6^	
ME (kcal/kg)	2900.00
CP (%)	16.81
Ca (%)	0.84
TP (%)	0.49
AP (%)	0.26
D-Lys	0.81
D-Met	0.32
D-Thr	0.55
D-Trp	0.20

^1^ Provided per gram of enzyme preparation: xylanase, 3500 units. ^2^ Provided per kilogram of bacitracin methylene disalicylate premix: bacitracin, 150 mg. ^3^ Provided per kilogram of preservatives: sodium diacetate, 1000 mg. ^4^ Provided per kilogram of diet: vitamin A, 5000 IU; vitamin D_3_, 400 IU; vitamin E, 10 IU; vitamin K_3_, 0.5 mg; vitamin B_1_, 2.0 mg; vitamin B_6_, 2.5 mg; vitamin B_12_, 0.02 mg; nicotinic acid, 55 mg; pantothenic acid, 10 mg; folic acid, 1.0 mg; and biotin, 0.1 mg. ^5^ Provided per kilogram of diet: 60 mg Fe (FeSO_4_⋅7H_2_O); 8 mg Cu (CuSO_4_⋅5H_2_O); 60 mg Zn (ZnSO_4_⋅7H_2_O); 50 mg Mn (MnSO_4_⋅H_2_O); 0.1 mg Se (Na_2_SeO_3_⋅5H_2_O); and 0.2 mg I (KI). ^6^ Values are calculated. ^7^ CON, basal diet; ME, metabolic energy; CP, crude protein; Ca, calcium; TP, total phosphorus; AP, available phosphorus.

**Table 2:** Compositions and nutrient contents of the experiment basal diets (air dry basis, %, Experiment 2).

Item	PC ^7^	LEP
Ingredients, %		
Maize	53.02	53.42
Wheat flour	5.00	5.00
Wheat bran	13.06	15.128
Soybean meal	9.40	7.30
Cottonseed meal	5.00	5.00
Distillers dried grains with solubles	10.00	10.00
Soybean oil	0.50	0.00
Calcium carbonate	1.60	1.61
Dicalcium phosphate	0.55	0.54
L-Lysine HCl	0.65	0.725
DL-Methionine	0.16	0.173
L-Threonine	0.11	0.129
L-Valine	0.00	0.025
NaCl	0.40	0.40
Preservatives ^1^	0.10	0.10
Montmorillonite premix ^2^	0.10	0.10
Antioxidants ^3^	0.05	0.05
Vitamin premix ^4^	0.10	0.10
Mineral premix ^5^	0.20	0.20
Total	100.00	100.00
Calculated Nutrients ^6^		
ME (kcal/kg)	2701.30	2747.20
CP (%)	16.28	16.80
Ca (%)	0.78	0.78
TP (%)	0.48	0.48
AP (%)	0.25	0.25
D-Lys	0.90	0.90
D-Met	0.41	0.41
D-Thr	0.59	0.59
D-Trp	0.14	0.14

^1^ Provided per kilogram of preservatives: sodium diacetate, 1000 mg. ^2^ Provided per kilogram of montmorillonite premix: montmorillonite, 850 g. ^3^ ENDOX^TM^ (Kemin industries Co., Ltd., Zhuhai, China). Butyl hydroxy anisd: 1.8%; Ethoxyquin: 2.7%.^4^ Provided per kilogram of diet: vitamin A, 5000 IU; vitamin D_3_, 400 IU; vitamin E, 10 IU; vitamin K_3_, 0.5 mg; vitamin B_1_, 2.0 mg; vitamin B_6_, 2.5 mg; vitamin B_12_, 0.02 mg; nicotinic acid, 55 mg; pantothenic acid, 10 mg; folic acid, 1.0 mg; and biotin, 0.1 mg. ^5^ Provided per kilogram of diet: 60 mg Fe (FeSO_4_⋅7H_2_O); 8 mg Cu (CuSO_4_⋅5H_2_O); 60 mg Zn (ZnSO_4_⋅7H_2_O); 50 mg Mn (MnSO_4_⋅H_2_O); 0.1 mg Se (Na_2_SeO_3_⋅5H_2_O); and 0.2 mg I (KI). ^6^ Values are calculated. ^7^ PC, basal diet; LEP, basal diet in low and protein levels.

**Table 3:** Effects of different protease or phytase on growth performance in ducks ^1^.

Item	CON ^2^	PA	PB	PTA	PTB	PTC	SEM ^3^	*p*-Value
Initial weight, g	519.11	519.11	519.11	519.15	519.18	519.17	23.0	0.90
Final weight, g	3175.11	3193.32	3198.34	3223.66	3200.01	3208.03	180.0	0.73
ADG, g/d	91.59	92.21	92.39	93.26	92.24	92.72	0.60	0.64
ADFI, g/d	188.60	190.78	194.18	188.72	192.59	189.16	2.74	0.53
FCR	2.06	2.07	2.10	2.02	2.08	2.04	0.04	0.45
Mortality, %	3.10	1.60	1.90	3.20	0.90	1.90	0.89	0.54

^1^ Means represent 7 pens of ducks with 120 birds per pen (*n* = 7). ^2^ CON, basal diet; PA, phytase A; PA, phytase B; PTA, protease A; PTB, protease B; PTC, protease C; ADG, average daily gain; ADFI, average daily feed intake; FCR, feed conversion ratio. ^3^ Standard error of the means.

**Table 4:** Effects of different protease or phytase on serum physiochemical parameters and digestive enzyme activities in ducks ^1^.

Items	CON ^2^	PA	PB	PTA	PTB	PTC	SEM ^3^	*p*-Value
Serum								
Phosphorus, mmol/L	2.01 ^a^	1.78 ^b^	1.85 ^a,b^	1.91 ^a,b^	1.95 ^a,b^	1.98 ^a,b^	0.10	0.04
Calcium, mmol/L	1.60 ^a^	1.41 ^b^	1.46 ^a,b^	1.55 ^a,b^	1.52 ^ab^	1.51 ^a,b^	0.09	0.04
Jejunal digesta								
Trypsin, units/mgprot	1406.73 ^b^	2022.20 ^a,b^	2056.65 ^a,b^	2766.32 ^a^	2447.23 ^a^	2213.54 ^a^	378.00	0.02
Chymotrypsin, units/mgprot	4.12 ^b^	4.48 ^a,b^	4.35 ^a,b^	6.11 ^a^	5.02 ^a,b^	6.38 ^a,b^	0.97	0.04

^a,b^ Means in a row with different letter differ (*p* < 0.05). ^1^ Means represent 7 pens of ducks with 4 birds per pen (*n* = 7). ^2^ CON, basal diet; PA, phytase A; PA, phytase B; PTA, protease A; PTB, protease B; PTC, protease C. ^3^ Standard error of the means.

**Table 5:** Effects of protease and phytase in low energy and protein diet on growth performance in ducks ^1^.

Items	PC ^2^	PCE	LEP	LEPE	SEM ^3^	*p* *	*p* ^#^	*p* ^+^
Initial weight, g	727.50	712.50	711.20	717.20	26.60	0.81	0.89	0.39
Final weight, g	3241.00 ^b^	3366.00 ^a^	3243.00 ^b^	3370.00 ^a^	73.23	0.20	0.04	0.19
ADG, g/d	89.78 ^b^	94.78 ^a^	90.40 ^b^	91.00 ^a^	2.85	0.19	0.02	0.08
ADFI, g/d	194.39	197.33	194.64	185.64	10.15	0.28	0.57	0.26
FCR	2.16	2.08	2.15	2.04	0.10	0.56	0.06	0.75
Mortality, %	1.84	2.14	3.29	3.00	0.19	0.29	0.99	0.78

^a,b^ Means in a row with different letter differ (*p* < 0.05). ^1^ Means represent 6 pens of ducks with 240 birds per pen (*n* = 6). ^2^ PC, basal diet; PCE, basal diet supplemented with enzymes; LEP, basal diet in low and protein levels; LEPE, basal diet in low and protein levels supplemented with enzymes. ^3^ Standard error of the means. *p* * Means nutrients levels effect. *p* ^#^ Means enzymes effect. *p* ^+^ Means nutrients levels × enzymes effect.

**Table 6:** Effects of protease and phytase in low energy and protein diet on serum physiochemical parameters and digestive enzyme activities in ducks ^1^.

Items	PC ^2^	PCE	LEP	LEPE	SEM ^3^	*p* *	*p* ^#^	*p ^+^*
Serum								
Glucose, mmol/L	12.22	11.61	11.70	11.80	2.59	0.86	0.79	0.72
Cholesterol, mmol/L	5.93	5.99	5.70	5.86	0.85	0.26	0.64	0.11
Low-density lipoprotein, mmol/L	0.67	0.76	0.76	0.83	0.15	0.13	0.13	0.90
High-density lipoprotein, mmol/L	1.63 ^b^	2.40 ^a^	1.72 ^b^	2.53 ^a^	0.60	0.51	0.01	0.90
Urea nitrogen, mmol/L	1.88	1.62	1.74	1.62	0.31	0.52	0.19	0.49
Phosphorus, mmol/L	2.11 ^b^	2.00 ^a^	2.12 ^b^	2.02 ^a^	0.13	0.81	0.03	0.98
Calcium, mmol/L	1.54 ^b^	1.45 ^a^	1.54 ^b^	1.45 ^a^	0.10	0.93	0.01	0.99
Aspartate aminotransferase, units/L	17.42	16.22	16.99	15.53	2.45	0.53	0.13	0.88
Alanine aminotransferase, units/L	13.31	11.77	12.91	12.37	0.60	0.97	0.65	0.83
Alkaline phosphatase, units/L	41.01	38.21	40.60	39.43	1.38	0.94	0.70	0.87
Jejunal digesta								
Trypsin, units/mgprot	1122.64 ^b^	1922.49 ^a^	1137.40 ^b^	1821.45 ^a^	46.83	0.68	0.01	0.58
Chymotrypsin, units/mgprot	2.55 ^b^	3.54 ^a^	2.47 ^b^	3.23 ^a^	0.44	0.57	0.01	0.86

^a,b^ Means in a row with different letter differ (*p* < 0.05). ^1^ Means represent 6 pens of ducks with 4 birds per pen (*n* = 6). ^2^ PC, basal diet; PCE, basal diet supplemented with enzymes; LEP, basal diet in low and protein levels; LEPE, basal diet in low and protein levels supplemented with enzymes. ^3^ Standard error of the means. *p* * Means nutrients levels effect. *p* ^#^ Means enzymes effect. *p* ^+^ Means nutrients levels × enzymes effect.
